# Robotic-Assisted Gait Training Combined with Multimodal Rehabilitation for Functional Recovery in Acute Dermatomyositis: A Case Report

**DOI:** 10.3390/brainsci15060650

**Published:** 2025-06-17

**Authors:** Wilmer Esparza, Rebeca Benalcazar-Aguilar, Gabriela Moreno-Andrade, Israel Vinueza-Fernández

**Affiliations:** 1 Facultad de Ciencias de la Salud y Bienestar Humano, Carrera de Medicina, Universidad Tecnológica Indoamérica, Ambato 180202, Ecuador; 2 Maestría en Neurorrehabilitación, Facultad de Posgrados de Ciencias de la Salud, Universidad de Las Américas (UDLA), Quito 170124, Ecuador; rebeca.benalcazar@udla.edu.ec (R.B.-A.); gabriela.moreno.andrade@udla.edu.ec (G.M.-A.); 3 Departamento de Fisioterapia, Hospital Carlos Andrade Marín, Quito 170136, Ecuador; 4 Center for Research on Health in Latin America (CISeAL), Pontificia Universidad Católica del Ecuador, Quito 170143, Ecuador; iavinuezaf@puce.edu.ec

**Keywords:** dermatomyositis, inflammatory myopathy, robotic rehabilitation, robotic-assisted therapy, Lokomat, gait training, functional recovery, neuromuscular rehabilitation, mobility, case report

## Abstract

This case report examines the impact of robotic-assisted therapy (Lokomat) on functional recovery in a 28-year-old male patient with acute dermatomyositis (DM), an autoimmune inflammatory myopathy causing progressive muscle weakness and disability. The patient underwent 21 sessions of robotic therapy combined with physical therapy, and occupational therapy over seven weeks. Assessments were conducted at baseline, week 10, and week 21 using standardized measures for balance, muscle strength, and functionality. Results demonstrated significant improvements across all domains: balance scores progressed from severe impairment (4/56 Berg, 0/28 Tinetti) to near-normal function (55/56, 24/28, respectively); muscle strength increased from grade 1/5 to 4/5 (MMT-8) in all tested muscle groups; and functionality improved from moderate dependence (59/126 FIM) to complete independence (126/126). The trunk functionality scores showed remarkable recovery from 12/100 to 100/100 (TCT), indicating restored trunk control. Lokomat-assisted therapy combined with conventional rehabilitation effectively improves proximal weakness and postural instability in DM. Robotic therapy enhances motor learning via repetitive movements and reduces therapist workload. Though limited by a single-case design, this study offers preliminary evidence for robotic rehabilitation in DM, previously unexplored. Controlled studies are needed to standardize protocols and validate results in larger cohorts. Advanced technologies show promise for functional recovery in inflammatory myopathies.

## 1. Introduction

Dermatomyositis (DM) is an idiopathic inflammatory and autoimmune myopathy that affects the skin and muscles, resulting in progressive weakness and disability [1]. Prevalence varies widely, with reports ranging from 5 to 32 cases per 100,000 individuals [2,3]. Incidence estimates suggest 7.98 cases per 1 million adults per year [4]. The disease is more common in women, with a 2:1 ratio, and typically manifests between the ages of 5 and 15 in children, and 40 and 60 in adults [5,6].

The disease primarily affects proximal and axial muscles that are crucial for posture, balance, and coordination [1,7,8]. This impairment is particularly evident in tasks requiring trunk stability and coordination, such as rising from a seated position or maintaining balance while walking [8,9]. Motor performance is undoubtedly impaired as seen in the reduction in both isometric and dynamic muscle strength. This is especially relevant in trunk muscles, which play a key role in core stability and overall muscular function [8].

Pharmacological treatment typically involves systemic corticosteroids, intravenous immunoglobulin, and immunosuppressive drugs, primarily to control inflammation [10]. Physical rehabilitation plays a vital role in functional recovery. Exercise-based interventions have proven beneficial in improving muscular coordination and reducing fatigue in patients with DM. Programs that include aerobic and strength training have shown positive outcomes not only in a functional capacity but also in quality of life [11,12,13]. Specifically, high-intensity strength training and resistance exercises have been effective in increasing muscle strength and improving physical function [11,14].

In recent years, multimodal rehabilitation programs, which integrate interventions such as physical therapy, occupational therapy, cognitive therapy, and more recently, robot-assisted therapy, have demonstrated significant positive effects on motor performance across various clinical populations. This comprehensive approach addresses multiple dimensions of motor control, including strength, coordination, proprioception, and functionality in daily living activities [15,16,17,18]. These programs offer a more holistic strategy to restore motor skills and independence, which is particularly relevant in complex neuromuscular conditions such as dermatomyositis.

In this context, the use of robotic devices has emerged as a promising alternative for treating such patients. These devices offer stability, coordination, and high-intensity training when required. Robotic devices have demonstrated increased efficiency and precision in rehabilitation. They can perform accurate exercise repetitions in a set period, optimizing therapy and reducing the cumbersome setup of traditional gait training [19]. Moreover, these devices improve motor function, gait speed, muscle tone, and mobility, particularly when therapy is early, intense, and prolonged [20]. In terms of cost, robotic gait training can lower staffing expenses by allowing one therapist to train multiple patients simultaneously using partial body weight support and robotic guidance to ensure safe and intensive training [21,22]. These devices also reduce the physical strain on therapists, enabling longer and more intensive sessions without overburdening staff [22,23]. When combined with conventional therapy, robotic therapy can accelerate functional recovery and enhance patient adherence to rehabilitation programs [16,24,25].

Despite these benefits, there are currently no studies that use robotic therapy in patients with DM. The use of robotic devices like the Lokomat has been explored in various neuromuscular conditions, showing benefits in gait and balance rehabilitation [24,26]. However, their effectiveness in DM patients remains under-researched and requires further scientific evidence [27]. Thus, the aim of this case study was to analyze the impact of robotic therapy Lokomat with orthosis on the functional recovery of an adult patient with acute DM. In this way, the patient’s progress in the Berg Balance Test, Tinetti Scale, strength Manual Muscle Testing-8 “MMT-8”, and functionality Functional Independence Measure “FIM”, Lieve Heyrman’s Trunk Control Test was assessed over 21 days of treatment.

## 2. Case Presentation

### 2.1. Anamnesis and Demographic Data

The case under study concerns a 28-year-old male patient residing in Puerto Quito, a town located approximately two and a half hours from the city of Quito. Prior to his current condition, the patient worked for nine years in a cookie factory as a packer and was exposed to various chemical products. He reported no relevant personal or family medical history. He completed secondary education and has no known drug allergies. He is fully vaccinated against COVID-19 (four doses). In December 2023, he was diagnosed with dermatomyositis (DM) at the Carlos Andrade Marín Hospital in Quito.

### 2.2. Reason for Consultation

The patient sought medical attention in December 2023 for a clinical condition with two months of evolution, characterized by progressive muscle weakness, abdominal pain, and erythema on the thighs, chest, abdomen, upper limbs, and face, accompanied by generalized muscle pain of moderate to severe intensity. This symptomatology significantly limited his mobility, preventing him from walking more than a few steps, even with assistance. He also presented with dysphagia, respiratory difficulty, edema in the left leg, face, and left arm, a fever of 37.8 °C, decreased appetite, and a weight loss of 8 kg over two months. Muscle weakness began in the lower limbs and progressed to the upper limbs, predominantly affecting proximal musculature, making it difficult for him to lift his arms and legs, requiring help with basic activities such as hygiene and dressing. Distal strength remained preserved.

### 2.3. Clinical Examination

During the clinical evaluation, osteotendinous areflexia was observed in the lower limbs and hyporeflexia in the upper limbs, with preserved superficial and deep sensitivity. Muscular hypotonia and signs of multisystem involvement associated with the disease were noted. Laboratory studies revealed elevated liver enzymes (three times above normal values), elevated myoglobin levels, and a creatine phosphokinase (CPK) level of 27,931 U/L, indicating significant muscle damage. Additionally, decreased adrenocorticotropic hormone (ACTH) levels were identified, which may have contributed to symptoms of hypoglycemia, weakness, fatigue, anorexia, abdominal pain, nausea, vomiting, diarrhea, fever, muscle and joint pain, and adrenal insufficiency.

### 2.4. Laboratory Tests and Diagnosis

To confirm the diagnosis of dermatomyositis and rule out other conditions, additional studies were performed, including immunological markers, TORCH panel, skin biopsy, specific antibodies for myositis, and electromyography. The results confirmed the diagnosis of DM and ruled out the presence of tumor markers. In addition, a cytolytic liver lesion likely of autoimmune origin was identified, with elevated TGO, AGP, ALT levels, and the presence of cytomegalovirus (IgM), prompting his referral to a specialized care center. Electromyography showed that the conduction velocities, latencies, and amplitudes in the median, ulnar, peroneal, and tibial nerves were within normal parameters for motor and sensory nerve conduction studies, including normal F-wave responses.

Further diagnostic studies included an abdominal ultrasound that revealed the presence of fluid in all quadrants, while the solid organs appeared within normal parameters; a venous Doppler ultrasound of the lower limbs showing normal findings with a competent superficial venous system; and a CT scan of the abdomen and thorax that showed no abnormalities in the bone structures or soft tissues. Additional laboratory tests—such as creatinine, urea, electrolytes, LDH, AST, ALT, GGT, alkaline phosphatase, uric acid, CRP, PCT, HIV, blood gases, STORCH, INR, glucose, and albumin—were performed to rule out infections like HIV and pulmonary tuberculosis.

Colonoscopy revealed thickening of the colon wall but no concerning lesions. Serological testing for hepatitis B and hepatitis C was positive for IgM and IgG, suggesting prior or ongoing infection.

These comprehensive evaluations, together with the clinical picture and the electromyographic results, supported the diagnosis of dermatomyositis while identifying potential comorbidities and ruling out alternative pathologies.

### 2.5. Pharmacological Treatment

Initial treatment included the administration of tramadol (50 mg) for pain management, metoclopramide (10 mg every 8 h) to control gastrointestinal symptoms, intramuscular corticosteroids, intravenous immunoglobulin (IVIG), prednisone, and omeprazole for gastric protection. Given his bedridden condition, physical and respiratory therapy was implemented, including ipratropium bromide (2 puffs every 8 h during the first weeks of hospitalization). Neurological physical rehabilitation therapy was also initiated, and the continuous monitoring of muscle involvement was performed via electromyography. Due to renal involvement, a nephrology consultation was requested, and nutritional support was provided to manage proteinuria and hypoproteinemia. Long-term treatment included omeprazole, prednisone, folic acid, calcibon D, and methotrexate. Given the presence of hepatitis B and C markers, antiviral therapy and hepatology evaluation were also considered for comprehensive management.

## 3. Assessments

Prior to conducting the assessments, the patient signed an informed consent form, and the intervention protocol was submitted to the Local Ethics Committee for approval (CODE: SGC-EI-INT.A.18.001). The intervention complied with the Helsinki Declaration regarding human participation in experiments (WMA Declaration of Helsinki, 2024).

**Figure 1:**
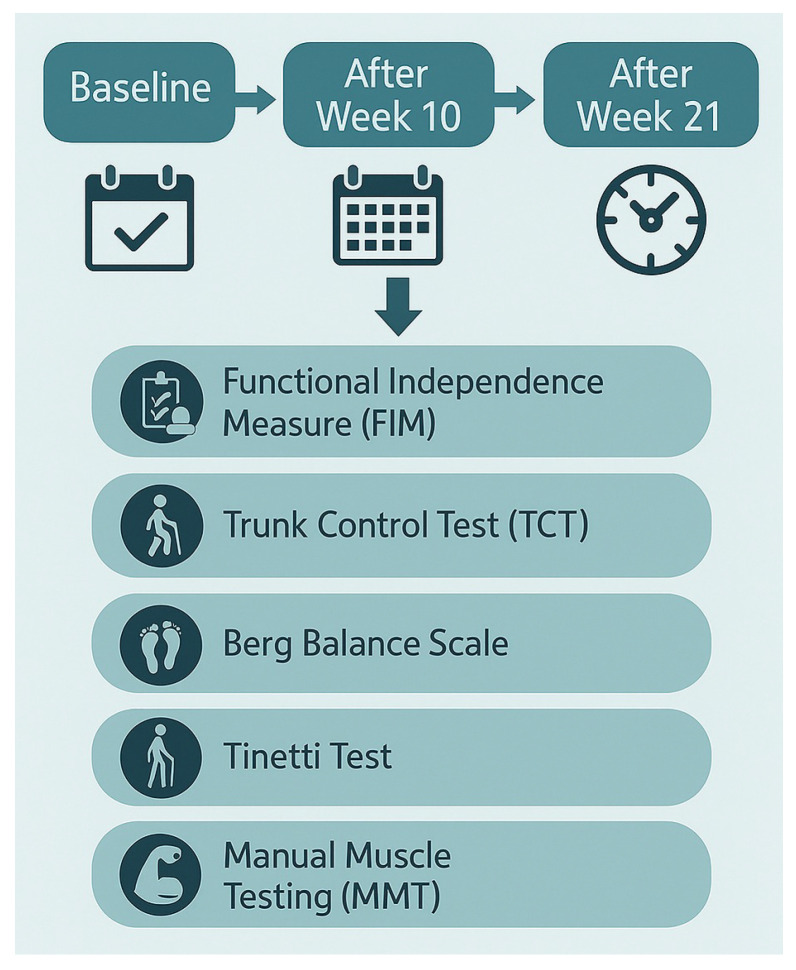
Assessment procedures and tests.

All functional assessments of the patient were performed in the hospital rehabilitation unit at three distinct time points, in accordance with the protocol detailed in Figure 1. Although the patient’s initial clinical condition posed challenges in obtaining responses for certain test items, all assessments were ultimately completed to establish comprehensive baseline measurements.

Below, we present a systematic overview of these assessment tools, summarizing their key features, scoring systems, and psychometric properties (reliability and validity) (Table 1). This structured format facilitates quick reference and highlights the core aspects of each measure.

## 4. Intervention

In January 2024, the patient began a conventional physical rehabilitation program during hospitalization (Pain management, contracture prevention, and maintenance of joint mobility). In February 2024, he was discharged and began treatment in March 2024 in the hospital’s outpatient rehabilitation area.

**Figure 2:**
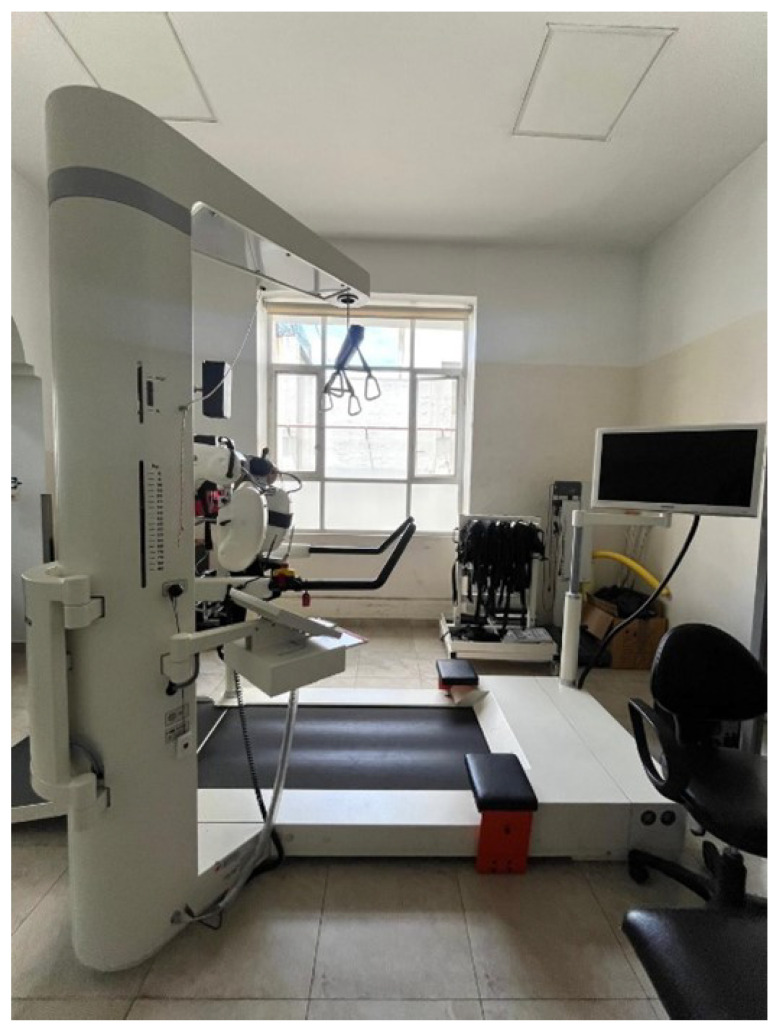
Lokomat device used for the robotic therapy.

The outpatient intervention focused on a comprehensive rehabilitation approach for an adult patient with acute dermatomyositis. A treatment protocol was implemented based on physical, robotic (Lokomat), and conventional therapy, in accordance with the clinical practice guidelines for gait recovery. For robotic therapy, the LokomatPro^®^ device manufactured by Hocoma AG Industriestrasse 4, 8604 Volketswil, Switzerland 2011, was used (Figure 2).

Robotic therapy began in March 2024, with a total of 21 sessions over seven weeks (3 sessions/week, 50 min each. Last session: 22 April 2024; Final assessment 25 April 2024). A physiotherapist experienced in using Lokomat^®^ followed the standardized protocol recommended by the manufacturer [47]. The protocol included three key phases.

### 4.1. Initial Phase: Measurement and Individualized Adjustments

The anthropometric parameters of the patient were recorded (height, weight, and lower limb length from the knee to the outer edge of the shoe), and measurements were taken for the placement of straps on the thigh (proximal to the knee), on the area distal to the knee (one hand below), and on the distal third of the leg (three fingers above the medial malleolus) [47]. Body weight support was initially set at 50% (considering 3 kg for the orthosis), and software programming was personalized based on neuromuscular impairment and evolved throughout treatment. However, strap and orthosis measurements remained fixed [47].

### 4.2. System Configuration Phase

Anthropometric data were entered into the Lokomat^®^ system, which generated guide values for orthosis adjustment and strap placement. A suspension harness was used to support the patient and facilitate progressive weight unloading [48].

### 4.3. Treatment and Progression Phase

Sessions 1–5: Basic biofeedback program (“The Path”). Sessions 6–18: Increased difficulty with advanced programs (“Win the Treasure”, “Find the Treasure”) and parallel bar work for postural control and weight-bearing training [49]. Session 19+: Transition to manual system (without orthosis), focusing on natural walking (up to 3.5 km/h), coordination, and speed [50]. The transition between modes depended on (1) improvement in functional capacity; (2) muscular strength evaluated by the system; and (3) the quality of the gait pattern [49].

**Figure 3:**
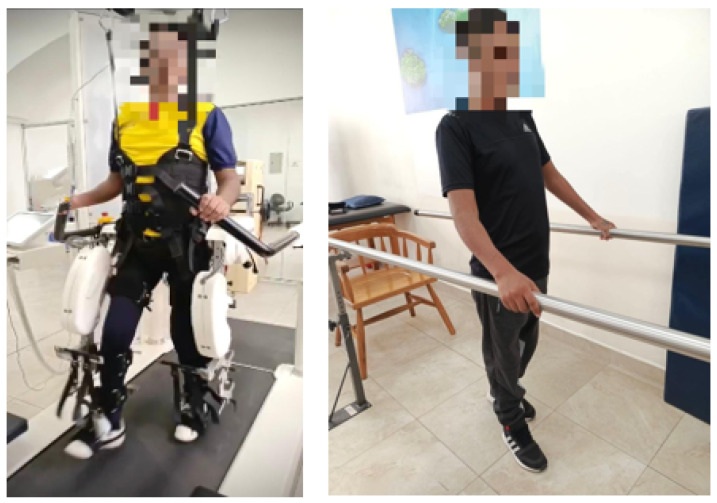
Robotic physical therapy and conventional training.

In parallel with robotic therapy, the patient received conventional neuromuscular rehabilitation program. This program included a comprehensive therapeutic plan focused on improving overall functionality, emphasizing mobility, postural control, and gait reeducation. Physical Therapy interventions (3 sessions/week “60 min/session”) involved bilateral active and assisted mobilization of shoulders, hips, hands, and feet to maintain joint range and prevent contractures, along with proprioceptive and exteroceptive stimulation in the lower limbs for postural control. Core strengthening, lumbopelvic dissociation exercises, and isometric work for pelvic and quadriceps activation were implemented, followed by gait training with parallel bars and a walker. Balance, coordination, and segmental strengthening of upper/lower limbs, abdomen, and lumbar muscles were addressed, alongside moderate aerobic training (stationary bike) and treadmill walking for endurance and gait symmetry. Finally, occupational therapy (3 sessions/week, 45 min/session) was focused on passive and assisted active mobility, coordination, strengthening (particularly upper limbs), compensatory strategies, and improving independence in daily living activities (Figure 3).

**Figure 4:**
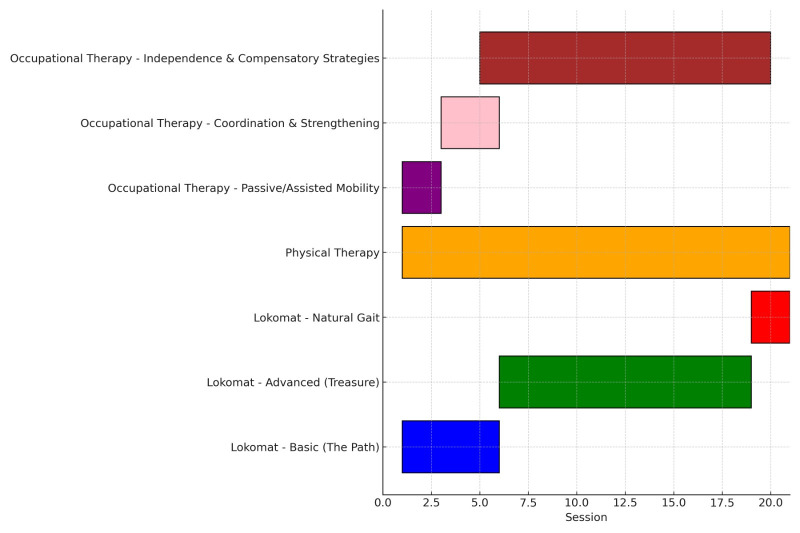
Timeline of multimodal therapeutic interventions.

Figure 4 delineates the chronological administration of multimodal therapeutic interventions throughout the treatment protocol.

## 5. Results

**Figure 5:**
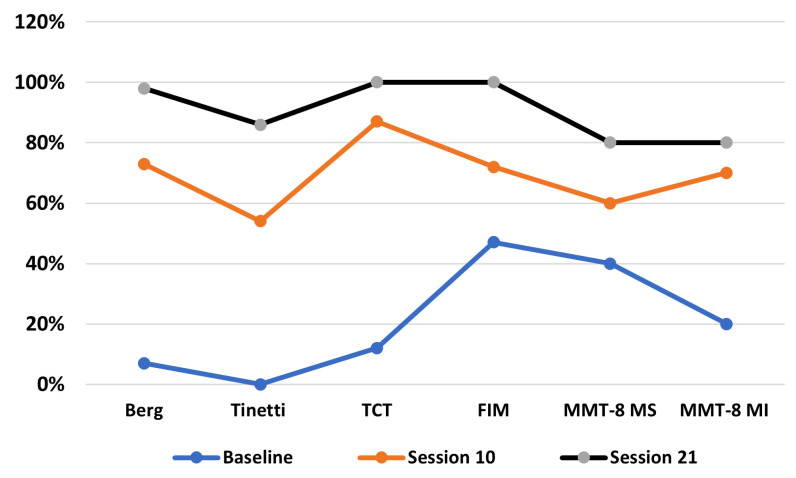
Percentage changes in the functional recovery.

The patient’s progress is presented descriptively through homogenized data represented as percentages to allow a clear visual interpretation (Figure 5). These data reveal the impact of the Lokomat intervention on key parameters such as balance, muscle strength, and overall functionality.

### 5.1. Balance

Results from the Berg and Tinetti scales demonstrate significant improvements in postural stability and gait. Initially, the patient exhibited severely impaired balance (4/56 in Berg and 0/28 in Tinetti), associated with a high risk of falls and total dependence for mobility.

In the second evaluation, a substantial improvement was observed (41/56 in Berg and 15/28 in Tinetti), indicating enhanced postural control and assisted gait. By the third evaluation, the patient achieved near-normal functionality (55/56 and 24/28), with independent and symmetrical gait.

### 5.2. Muscle Strength

At baseline, the patient presented severe muscle weakness (1/5 in lower limbs and proximal upper limbs), limiting basic functions. Progress tracked through the EMMT-8 scale showed functional gains by the second evaluation (up to 4/5 in various muscle groups), enabling transfers and object manipulation. By the third evaluation, the patient reached 4/5 bilaterally across all segments, supporting the independent performance of daily activities.

### 5.3. Functionality

Functionality assessed via TCT and FIM revealed significant improvements. The patient progressed from severely impaired trunk control (12/100 on TCT) to full control (100/100) by the end of treatment, indicating the effective activation of axial muscles. FIM scores also improved substantially: from moderate dependence (59/126) to complete independence (126/126).

The patient functional progression across 21 therapy sessions revealed a clear, time-dependent improvement in balance, trunk control, strength, and overall independence. At baseline, the patient exhibited severely compromised functionality, with scores of 7% (Berg), 0% (Tinetti), and 12% (TCT). By session 10, these measures improved substantially—Berg (73%), Tinetti (54%), and TCT (87%)—coinciding with gains in muscle strength in both upper (MMT-8 MS: 60%) and lower limbs (MMT-8 MI: 70%). Functional independence also rose markedly (FIM: 72%). By session 21, the patient reached near or complete normalization in all scales—Berg (98%), Tinetti (86%), TCT and FIM (100%)—reflecting a strong correlation between balance recovery, muscle reconditioning (MMT-8 MS/MI: 80%), and regained autonomy.

## 6. Discussion

This case report demonstrates substantial functional improvements in a young adult patient with acute DM following a combined rehabilitation approach that integrated robot-assisted gait training (RAGT) and traditional physical and occupational therapy. Improvements were particularly evident in balance (Berg and Tinetti scores), trunk control (TCT), muscle strength (MMT-8), and independence in daily living activities (FIM). These findings are consistent with previous studies that underscore the benefits of multimodal rehabilitation for neuromuscular recovery [15,16,17,18]. Although robotic therapy has not been extensively studied in dermatomyositis, the outcomes align with evidence from stroke and muscular dystrophy rehabilitation, suggesting that Lokomat-based therapy can significantly accelerate recovery and reduce disability [6,20,24].

The initial severely impaired balance (4/56 in Berg and 0/28 in Tinetti) is consistent with the literature indicating that proximal muscle weakness characteristic of dermatomyositis severely compromises safe ambulation [51]. The improvements observed in postural control and gait align with studies supporting robotic therapy in neuromuscular disorders, where repetitive and guided training using Lokomat promotes motor learning and neuromuscular reorganization [52,53,54,55].

The baseline severe muscle weakness (1/5 in lower limbs and proximal upper limbs) is typical in dermatomyositis, especially in proximal muscles, and directly correlates with functional disability [56]. The functional gains observed (up to 4/5 in various muscle groups) are consistent with evidence that robotic devices enhance both muscle strength and neuromotor control [25,53].

The progression from severely impaired trunk control (12/100 on TCT) to full control (100/100) supports the findings by Chirila et al. [56], who emphasize axial impairment as a key factor in balance limitations in DM. The substantial improvement in FIM scores (from 59/126 to 126/126) reflects not only regained strength and motor control but also a comprehensive improvement in quality of life. The literature supports this, showing that robotic rehabilitation can accelerate reintegration and social participation [52,53].

The functional recovery observed not only encompasses the motor aspects but also translates into a comprehensive enhancement of the patient’s quality of life, enabling personal independence and social reintegration. This highlights the need to incorporate advanced technologies like Lokomat into rehabilitation protocols for severe inflammatory muscle diseases.

## 7. Limitations

This study presents a key methodological limitation: it involves a single patient and lacks a control group that received only conventional therapy. As such, it is not possible to isolate the effect of the robotic therapy from that of the conventional therapies or their potential synergistic interaction. This severely limits causal inference. It is therefore critical to emphasize that the observed gains should be attributed to the integrated intervention model, not solely to the robotic component.

In the case described, robotic therapy and conventional therapy were conducted in parallel, raising an important question for clinical interpretation: To what extent does the robotic component accelerate or amplify recovery compared to traditional rehabilitation alone? While existing evidence suggests that RAGT provides enhanced intensity, repeatability, and patient engagement [15,21], the specific magnitude of its contribution remains undetermined in this case. For example, in patients with chronic stroke, studies have demonstrated that exoskeleton-assisted gait training can significantly improve walking speed and endurance compared to conventional gait training alone [57]. These findings underscore the potential of robotic devices to enhance motor recovery beyond the capabilities of manual therapy, highlighting the importance of carefully quantifying their effects in different pathologies and patient profiles.

Micera et al. [15] highlight that for neurotechnologies to deliver clinically meaningful improvements, their use must be both personalized and mechanistically informed, taking into account patient-specific responsiveness. In our case, the patient demonstrated early responsiveness to RAGT, which may suggest a favorable profile for technology-assisted recovery. Nevertheless, future research should focus on controlled clinical trials comparing conventional, robotic, and combined therapy models, and should include biomarkers or physiological data to stratify patient response and elucidate mechanisms of action [15,58].

Moreover, it remains uncertain whether the improvements were the result of neuroplastic changes induced by high-intensity robotic training, the motivational aspects of interactive biofeedback, or the synergy of task-specific physical and occupational training. This underscores the need for multimodal assessment frameworks that combine behavioral, neurophysiological, and biomechanical data to evaluate rehabilitation outcomes comprehensively. Furthermore, it underscores the importance of proposing multicenter, large-sample studies aimed at validating the effectiveness of these interventions and guiding future targeted research initiatives.

Another relevant limitation that must be acknowledged is the influence of patient-specific characteristics, such as age. In this case, the participant was 28 years old, which likely contributed positively to the rehabilitation outcome. Younger adults tend to have greater neuroplastic potential, faster metabolic recovery, and higher levels of physical resilience [59]. Additionally, psychological factors such as motivation, adherence, and self-efficacy are generally more favorable in younger populations [60]. Therefore, it is plausible that the improvements observed are partially attributable to the patient’s age-related advantages, which may not generalize to older or less physically resilient individuals. This highlights the need for caution in extrapolating these findings across different age groups and supports the importance of age-stratified analyses in future studies.

## 8. Conclusions

This case report demonstrates that the integration of Lokomat-assisted robotic therapy with conventional physical and occupational therapy was associated with substantial improvements in balance, muscle strength, trunk control, and functional independence in a young adult patient with acute dermatomyositis. Over the course of 21 sessions, the patient progressed from severe disability to full independence in daily living activities.

Objective outcomes (Berg Balance Test, Tinetti Scale, EMMT-8, TCT, and FIM) confirmed the significant and progressive recovery of motor and functional abilities.

However, it is essential to acknowledge that the single-case nature of this study, without a control group, limits the ability to establish causality or isolate the specific contribution of robotic-assisted training from traditional therapy or their potential synergy. Furthermore, the observed improvements may also be partly attributable to the patient’s relatively young age, which likely favored faster neuroplastic adaptation and physical resilience.

These limitations highlight the need for caution in generalizing these findings to broader or older patient populations. To strengthen the evidence base, future studies should adopt multicenter, controlled designs comparing robotic-assisted therapy, traditional rehabilitation, and combined approaches. Such trials should also incorporate neurophysiological, biomechanical, and behavioral assessments to fully capture the impact of these interventions and identify patient-specific predictors of responsiveness.

Finally, while these preliminary results support the potential role of robotic-assisted therapy as a complement to conventional rehabilitation in dermatomyositis, further rigorous studies are required to confirm its effectiveness and optimize therapeutic strategies for this rare and disabling condition.

## Figures and Tables

**Table 1:** Assessment domains, tools, score ranges, key features, and measurement properties.

Domain	Tool	Score Range	Key Features	Reliability & Validity
Balance	Berg Balance Scale (BBS)	0–56 (Higher = better balance)	14 tasks assessing static and dynamic balance [28,29].	High reliability (ICC: 0.97–0.99); high internal consistency (Cronbach’s alpha: 0.886–0.923 [30,31,32,33].
Balance	Tinetti Test (POMA)	0–28 (Higher = better balance/gait)	Balance and gait subscales; assesses fall risk [34,35].	High inter-rater reliability (ICC ≥ 0.90); moderate-to-good validity; predictive validity for falls [36,37].
Muscle Strength	Manual Muscle Testing 8 (MMT-8)	0–5 (Higher = normal strength)	8 muscle groups; resistance-based assessment [38].	High internal reliability (Spearman: 0.91–0.98); subject to examiner variability [38,39].
Functionality	Functional Independence Measure (FIM)	18–126 (Higher = more independence)	18 items (13 physical, 5 cognitive); 7-level scale assessing ADL [40,41].	High internal consistency; strong inter-rater reliability; validated across diverse population [42,43].
Trunk Control	Trunk Control Test (TCT)	0–100 (Higher = better trunk control)	4 tasks assessing trunk performance in different positions [44].	High ICC (0.979); significant correlations with other motor function tests [45,46].

## Data Availability

The original contributions presented in this study are included in the article. Further inquiries can be directed to the corresponding author.

## Abbreviations

The following abbreviations are used in this manuscript:

ADLs	Activities of Daily Living
ALS	Amyotrophic Lateral Sclerosis
BBS	Berg Balance Scale
DM	Dermatomyositis
FIM	Functional Independence Measure
ICC	Intraclass Correlation Coefficients
MMT	Manual Muscle Testing
MMT-8	Manual Muscle Testing of 8 muscle groups
POMA	Performance-Oriented Mobility Assessment
TCT	Trunk Control Test
TUG	Timed Up and Go
